# The ‘everyday work’ of living with multimorbidity in socioeconomically deprived areas of Scotland

**DOI:** 10.15256/joc.2014.4.32

**Published:** 2014-05-28

**Authors:** Rosaleen O’Brien, Sally Wyke, Graham G.C.M. Watt, Bruce Guthrie, Stewart W. Mercer

**Affiliations:** ^1^General Practice and Primary Care, Institute of Health and Wellbeing, University of Glasgow, Glasgow, UK; ^2^College of Social Science, Institute of Health and Wellbeing, University of Glasgow, Glasgow, UK; ^3^Quality, Safety and Informatics Research Group, University of Dundee, Dundee, UK

**Keywords:** multimorbidity, complexity, self-management, chronic illness, quality of life, mental health, primary care, qualitative

## Abstract

**Background:**

Multimorbidity is common in patients living in areas of high socioeconomic deprivation and is associated with poor quality of life, but the reasons behind this are not clear. Exploring the ‘everyday life work’ of patients may reveal important barriers to self-management and wellbeing.

**Objective:**

To investigate the relationship between the management of multimorbidity and ‘everyday life work’ in patients living in areas of high socioeconomic deprivation in Scotland, as part of a programme of work on multimorbidity and deprivation.

**Design:**

Qualitative study: individual semi-structured interviews of 14 patients (8 women and 6 men) living in deprived areas with multimorbidity, exploring how they manage. Analysis was continuous and iterative. We report the findings in relation to everyday life work.

**Results:**

The in-depth analysis revealed four key themes: (i) the symbolic significance of everyday life work to evidence the work of being ‘normal’; (ii) the usefulness of everyday life work in managing symptoms; (iii) the impact that mental health problems had on everyday life work; and (iv) issues around accepting help for everyday life tasks. Overall, most struggled with the amount of work required to establish a sense of normalcy in their everyday lives, especially in those with mental–physical multimorbidity.

**Conclusions:**

Everyday life work is an important component of self-management in patients with multimorbidity in deprived areas, and is commonly impaired, especially in those with mental health problems. Interventions to improve self-management support for patients living with multimorbidity may benefit from an understanding of the role of everyday life work.

Journal of Comorbidity 2014;4:1–10

## Introduction

Multimorbidity, defined as two or more chronic, long-term, conditions [1]), is common, increases with age, and is socially patterned, with a much higher prevalence in areas of high socioeconomic deprivation [2]. Multimorbidity begins some 10–15 years earlier in the most-deprived areas, compared with the least-deprived areas [2]. Some of the most complex combinations of conditions to manage are those that include both mental and physical health conditions [3], and the prevalence of depressive symptoms is associated with the number of physical conditions, in a ‘dose–response’ relationship [4, 5]. Deprivation exacerbates this relationship, and thus, as the number of physical conditions increases within individuals, the prevalence of associated mental health problems is higher as deprivation increases [2]. Multimorbidity is also associated with lower quality of life [6, 7], and this relationship is again exacerbated by social deprivation [8]. Previous qualitative work conducted with general practitioners (GPs) and practice nurses in the top 15% most deprived areas of Scotland, UK, illustrated how difficult it is, at least in the context of their practice, to disentangle the complex relationship between physical health, mental health, and social problems [9].

Qualitative research on chronic illness has largely focused on capturing and describing the inordinate amount of ‘work’ [10] that illness management creates for individuals [11]. Critical self-care decisions have to be made in the context of daily life [12], which can give rise to tensions and moral dilemmas through efforts to normalize daily life and live ‘well’ [13]. Major changes to everyday life, consequent on illness, can also present ‘identity dilemmas’ [14] or prompt ‘biographical disruption’ [15, 16], which also have to be managed. The management of multimorbidity may be particularly disruptive to daily life as it can necessitate regular attendance at multiple appointments [17], taking many medications at different times [18], and following (sometimes conflicting) advice from GPs regarding the optimal management of each condition (this has led to calls for ‘minimally disruptive medicine’ that minimizes the burden of treatment in the context of an individual’s life and wider context) [19]. According to Corbin and Strauss, managing chronic illness, or any combination of chronic illnesses, relies on performing ‘three lines of work’, which organize the various tasks relating to managing illness: 1. Illness work, for example, symptom management, self-diagnosis, crisis prevention and management, and following a recommended care plan; Everyday life work, including going about life, performing housework, engaging in paid employment, maintaining relationships, etc; and 3. Biographical work, such as re-negotiating identity in light of illness(es) [10]. Recent work with health professionals has suggested that patients with a complex mix of personal, social, and medical problems may also lack the personal, social, and material resources to manage ‘well’ [9, 20]. This suggests that the everyday life work that patients with multimorbidity may have to perform in comparable settings, might (i) have a deleterious effect on their overall wellbeing and (ii) make it more difficult for GPs to help them [9].

Evidence suggests that the burden of managing multimorbidity is likely to be exacerbated both by depressive symptoms, and by living in areas of high socioeconomic deprivation, where people have fewer personal, social, and material resources to draw on [9, 21, 22]. Yet, this is a population that may benefit the most from targeted support for self-management [23, 24]. We need more detailed insights into the ways individuals currently manage multimorbidity in relation to other problems (e.g., mental health problems and social problems associated with deprivation). This qualitative study is part of a programme of research on patients living in deprived areas of Scotland with multimorbidity, which seeks to understand the needs of such patients in order to develop a primary care-based complex intervention to help patients ‘live well’ with multimorbidity [2, 9].

The overall aim of the qualitative study was to investigate the ways in which people living in areas of high socioeconomic deprivation managed multimorbidity. Participants’ descriptions of their performances of everyday life work (which was one of several substantive areas identified through analysis of accounts of managing multimorbidity) highlighted what they appeared to struggle with, and what was perceived to be managed ‘well’. The analysis presented in this paper thus focuses on everyday life work. Findings from other substantive areas will be published separately.

## Method

This qualitative study was conducted in Year 1 of a 4-year programme of research (2009–2013) designed to develop and evaluate a primary care-based, intervention that aimed to enhance wellbeing of individuals with multimorbidity. This ‘baseline’ qualitative study was conducted to help understand the issues facing patients with multimorbidity in deprived areas, and was done in conjunction with other studies which sought to quantify the problem of multimorbidity in relation to deprivation [2], and understand the pressures faced by primary care staff [9]. The final intervention, which will be reported at a later date, targeted younger patients (aged 30–64 years), in deprived areas (practices had a high percentage of patients in the top 15% most deprived areas in Scotland; see [9] for discussion), who were identified by their GPs as being in need of greater support (GPs were instructed to recruit patients based on these criteria). The suitability of the target group was confirmed through a practice-based prevalence study in areas of high deprivation (two practices) and low deprivation (two practices), which included case note review, and analysis of national primary care data which mapped out multimorbidity in the Scottish population by age and deprivation [2].

GPs were asked to help select patients for interview and varied in their approach to identifying patients. Some based their selection of patients through counting the number of conditions an individual had (identified through practice disease registers). However, most GPs reported that existing disease registers were not able to capture the complexity of multimorbidity they dealt with in practice. They reported that, in the context of deprivation, problems such as chronic pain and addictions were common, but never included in practice registers. Most GPs based their selection of patients on a combination of counts of conditions, knowledge of wider problems, and their experience of working with particular patients (see [9] for more detailed discussion on recruitment). We noted that this approach to recruitment appeared to identify people with intractable problems (seven participants were interviewed who were identified in this way). To ensure we included a wider range of views on managing multimorbidity, we used case notes to purposively sample a further seven participants with a range of combinations of problems (e.g., conditions with or without identified mental health problems; with or without social problems). We carefully reviewed the sample to ensure we included participants who had a combination of problems, of different ages (to ensure an even spread of those in their 40s, 50s, and 60s) and a balance in terms of sex (see Table 1 for details of the sample). Contact with participants was approved by the West of Scotland Research Ethics Committee REC 08/S0701/120.

We over-recruited for the interview study (the challenges of conducting research with vulnerable groups have been well documented, for example see [25]). Overall, 30 patients were recruited initially (one withdrew). We had aimed to interview approximately 20 participants in total. Considerable efforts were needed to cultivate research relationships throughout the duration of fieldwork. Ill health, and often difficult circumstances (e.g., sudden bereavement, family problems, transience), made it difficult for many to agree to, or keep, interview appointments. Participants who suffered from depression understandably found it difficult to keep appointments if they were feeling particularly unwell on the day of interview (it was not uncommon for the researcher to attend appointments on a number of occasions and not be able to gain access). Recruitment therefore involved regular telephone discussions with participants over weeks, sometimes months, prior to the interview. These provided insights into the ways participants’ everyday lives were affected by a range of problems. This was documented in field notes and later explored within recorded interviews when possible. Fourteen patients (8 women and 6 men) were eventually interviewed.

The interview topic guide was designed to explore how participants defined their current problems as they viewed them, and to explore how they were currently managing. This was based on previous qualitative work we conducted with GPs in deprived areas, which revealed a deeper and broader picture of multimorbidity, one that included social complexity, which contrasted with the clinical count of conditions ordinarily captured by epidemiologists [9]. We left it open for participants, at the start of interview, to define what their ‘main problems’ were and accounts were then probed (See Box A for topic guide). We asked about any additional problems, formally recorded by their doctor, if they were not raised by the participants (we had obtained permission to access case notes). It was our intention to gain insight into how participants perceived and managed their problems. This sometimes differed from how their doctors may have viewed them.

Most interviews took place in the participants’ homes, but two were conducted in a private meeting room at their local general practice. All interviews were digitally recorded with participants’ written consent, and had an average duration of 80 min (ranging from 45 to 180 min). RO also wrote field notes immediately after each interview, which summarized the context and noted initial analytic ideas.

Interviews were transcribed verbatim and transcripts cross-checked for accuracy by the first author (RO). NVivo 9 software (QSR International Pty Ltd) was used for data management. The process of data analysis was continuous and iterative, typical of an adapted grounded theory approach [26]. Half of the transcripts were coded by RO under broad themes derived from: (i) research questions, (ii) emergent themes identified during fieldwork and recorded in field notes, and (iii) themes that were identified *in vivo* through the process of coding. The co-authors SW and SM then coded several transcripts independently and a workable coding framework was agreed on. The principal categories for coding the data as a whole were as follows:

1. How is multimorbidity being defined?
2. What are the current strategies being used to manage multimorbidity?
3. Participants’ ideas about what would help (individual/system)
4. Ideas on good outcomes.

SW and SM also cross-checked data coded under selected categories for quality purposes.

The development of our analytic focus on everyday life work began with RO recording in field notes that there appeared to be implicit management ‘work’ going on. For example, it was striking that frequent references were made to the amount of housework being performed by people who described severe limitations due to pain, physical disabilities, and mood swings. References were also made to the work of managing the effects of deprivation, for example, the hard work involved in simply getting through the day because they lived in an area they found depressing, with few resources (note we did not ask participants *explicitly* about their experiences of living in very deprived areas, but these were an important reference point within their accounts).

When coding commenced, we noted that participants described distinct areas of ‘work’ that was being undertaken to manage medical, social, and psychological problems (e.g., “taking all of my medication is a full-time job”). This corresponded well with Corbin and Strauss’ analysis of the ‘three lines of work’ (illness, biographical, and everyday life work) [10], and so we examined our data in relation to these three areas. We focus on everyday life work as this has been relatively unexplored in comparison to illness and biographical work. Our strategy was to assign the code ‘everyday life work’ to anything interpreted as relating to the ‘everyday tasks of life’ [10] described by participants. In our sample this included participants’ descriptions of paid work; housework; self-care; care of others; relationships (which were sometimes described as difficult and involved ‘work’ or emotional ‘labour’); time management; efforts to manage social problems, such as financial, housing, or neighbour disputes; and work involved in negotiating the physical space of the areas they inhabited (e.g., having to struggle uphill to get a bus, having to avoid certain areas and certain people).

## Results

The overall aim of the baseline qualitative study was to explore the ways in which people living in areas of high socioeconomic deprivation manage multimorbidity. In this paper, we focus on the relationship between everyday life work and multimorbidity. Participants’ descriptions of their performances of everyday life work serve to highlight the contexts in which they struggled and others they felt they could manage ‘well’. Four main themes about everyday life work were identified in the analysis and could be used to characterize responses: (i) the symbolic significance of everyday life work in relation to being able to establish some kind of ‘normal life’; (ii) using everyday life work to manage symptoms; (iii) how mental health problems impaired everyday life work; and (iv) accepting help for everyday life tasks. Participants’ accounts of everyday life work illustrate some of the things that can help, or hinder, the effective management of multimorbidity and affect the general wellbeing of these patients.

### The symbolic significance of everyday work

Housework or taking care of a public space around their home (such as a garden) was commonly presented, particularly by women, as work they felt compelled to do, regardless of the difficulties or pain it caused (see Table 2 for description of participants’ problems). One respondent described struggling on with housework despite the “layers of pain” she often had to manage: “I’ve got to be doing something. I’ve got to be tidying up. I’ve got to make sure my house is the way I want. No I wouldn’t sit in my own filth, no. I’ve got to do my housework whether it kills me or not.” (P4 B: female, 50 years, multimorbidity [MM] + depression as well as diabetic neuropathy and osteoporosis).

Another participant described how she would have to spend “hours each morning slowly trying to get my body moving” (due to osteoarthritis and depression, among other conditions, see Table 2) and that this meant she was unable to participate fully in a course she had enrolled in. However, she emphasized how important it was to be seen performing work around the home: “doing the garden nearly killed me [laughing], but I had to get it done” (P10 B: female, 64 years, MM + depression).

Both male and female participants expressed concerns that they might appear “idle” (P12 B: male, 52 years, MM + depression) if they did not occupy themselves with chores around the home, and emphasized repeatedly their desire to engage in paid employment (even though it seemed unlikely that most would ever work again) “I’ve worked all my life” (P1 B: male, 53 years, MM + depression), “I’ve always worked” (P7 A, male, 54 years, MM + depression), and “I want to work” (P4 B).

There was suggestion that, for some, work around the home underlined their moral worthiness and enabled them to appear, and also believe, that they were managing ‘well’ in spite of illness and limited material resources. Cleanliness (in terms of both physical space and morals) appeared to have particular significance in the context of poverty as it seemed to be an important way of demonstrating that they were not ‘dirty’ (other researchers have highlighted this link between poverty and dirt; indeed, this is culturally rooted in everyday language, e.g., ‘dirt poor’): “I keep things clean. My house is clean. It might be poor, but it’s absolutely clean. You could eat your dinner out of my toilet” (P2 A: female, 62 years, MM + depression).

Participants’ accounts of everyday work around the home (such as cooking, cleaning, and gardening) suggested that these activities symbolized to them that they were “get(ting) on with it [illness]” (P7 A) and working hard, “knuckling down” (P4 B), to establish some kind of normalcy within their day.

### The importance of everyday tasks to manage emotional distress

The tasks of everyday life work appeared to serve an important function, for some, in helping to manage the changes to their lives that came with their illness, for example, boredom and isolation following loss of paid work and having lives very different from other people in their age group. Most accounts emphasized the positive impact of *doing* things, even housework, for managing emotional distress that arose as a result of these losses. *Not doing*, as a consequence of physical disability relating to having a number of debilitating conditions, in the sense of “just sitting and vegetating” (P12 B), was also commonly identified as prompting or exacerbating bouts of depression, which also had to be managed.

One participant who had suffered a spinal injury at work and was in pain much of the time, said he had to work very hard to stay mentally well (he had suffered from depression since his accident, largely due to the loss of paid employment and his change in circumstances) (P12 B, Table 2). He described how finding ways of staying occupied and active helped to prevent a “mental death”. However, he said that social resources in his area were scarce and this made it difficult to find activities that might distract him and help him to stay psychologically well: “I’m trying to think of other things to do [in the area] and I can’t think of anything… There’s nowhere round here. There’s nothing. There’s no… Like I said there’s no Leisure Centre you could go to… and they closed the Community Centre” (P12 B).

For another participant, housework was perceived to be an important distraction, which was perceived to help manage difficult emotions. She described how she struggled to make sense of the changes that her many illnesses had brought. When she was able to be active around the home, she found it helped distract her from feelings of anger (that her life so little resembled that of her peers), grief (that her illness had resulted in so many losses, particularly paid employment which had been an important part of her identity), and fear (about her health and how it would impact everyday life work in future). However, she described how she still had “bad days” (in terms of pain) when she was simply unable to engage with everyday activities. She described how her mental health would deteriorate when she was less able to continue ‘doing’: “Sometimes I sit and cry. I do. I sit and cry ’cause I think ‘God’s sake you’re only 50 years of age. How did it come to this that you’re in so much pain? That you’ve got all these things you’ve got to do? And you’ve got all these illnesses. Just one after the other’, you know, ‘everything seems to be happening’. I can’t make sense of it” (P4 B).

Everyday life work appeared to serve a particular function for some who were managing difficult emotions. Although the majority who raised this in interview suffered from depression, there was no simple relationship between emotion management and depression. Rather, the emotions that participants struggled with appeared to be closely related to the consequences of managing multiple problems, which were perceived to limit their lives at relatively young ages (commonly resulting in having to give up employment and experiencing social isolation). The role of social context, in particular the availability of local resources, to help manage the psychological consequences of multimorbidity (with or without depression) is also underlined.

### Difficulties keeping going with everyday life work

In contrast to those for whom everyday tasks were symbolic of managing well or helped with emotional distress, some accounts illustrated how hard it was to keep going with everyday life work in the midst of struggling with multimorbidity and depressive symptoms (see Table 2 for an example of a man feeling he cannot undertake everyday work). For example, one man felt that life had come to a standstill and “every day, week, month, year is the same”. He had been unemployed for over 10 years and had long forgotten what it was like to have meaningful everyday activities to fill his day; his only distraction, which he took up when he became so distressed that he wanted to kill himself, was “jumping in the car” and driving away from his local area. However, even these “escapes” (apparently from his own life) were described as repetitive and inescapable chores: “I just drive the same sort of circle. I wouldnae [would not] go out [of] that circle” (P11 B: male, 52 years, MM + depression).

Other participants described how day-to-day routines they had previously relied upon “went out the window” (P1 B), when they had become depressed. For one person, the stress of her mother’s illness made it very difficult to include daily activities that might help her manage, or improve, her mental health. Everyday life work had seemingly become focused on going “up and down to my ma night and day” and she described receiving little or no support from family and friends (P2 A). She felt that both her physical and mental health had deteriorated as a consequence, and described the burden she felt: “If she [her mother] dies if would be… It’d be a help. I know it’s an awful thing to say but that’d be such a weight off my… a burden off me, you know. I could maybe think about myself” (P2 A).

Another participant who had suffered with chronic depression, along with diabetes, expressed a hope that he might get life “started” again, but had difficulty “finding the motivation”, particularly in “putting the plan [e.g., to exercise more] into action” (P1 B). He had found attending a self-management support group for a short period had been helpful: “It’s encouragement… all the doctors were shaking your hands and these football players came up…saying ‘come on! You can do it’… You felt good within yourself” (P1 B). However, as he lived a relatively isolated life, he had struggled with motivation once the intervention ended.

Implicit in these accounts is the impact of living in a ‘poor area’ with few resources. For example, P11 B, whose experience is described above, told of having to confront, on a daily basis, a visibly “poor area” and live in “awful housing”. Thus the everyday life work in patients with multimorbidity, who described the particular impact of depression on their lives, was evidently made much harder by their socioeconomic circumstances (P11 B) (see also Table 2).

### Accepting help to support everyday life work

A final way in which everyday life work was discussed was in the context of *not* accepting help with everyday life work. This appeared to be related to a need to manage moral identities and maintain continuity of self-identity of independence, strength, and self-reliance.

Participants who experienced difficulties performing everyday life work (e.g., housework, personal hygiene, doing shopping, seeing friends) due to mobility issues, were usually offered aids (e.g., walking sticks and mobility scooters). However, their public use was discussed as fraught with challenges to personal and moral identities, explaining why there was reluctance to take up them up. Some felt they may be perceived as ‘dependent’, ‘lesser’ and ‘morally unworthy’ of receiving greater support, e.g., if they used sticks in public (see interview extract in Table 2).

Other participants described how they would rather maintain a continuous sense of their personal identity rather than ask for, or accept, help. One woman who suffered from osteoarthritis along with diabetes, asthma, and depression emphasized how her deteriorating mobility was ruining her life: “I cannae [cannot] get out. I cannae live my life if I cannae walk” (P2 A). However, she was reluctant to take up any offers of help, such as the suggested use of a mobility scooter “I’m not getting on one of them!” (P2 A), because she perceived this to be as a threat to her identity as a “strong” woman who “always coped” and “never asked for help”. She said: “I’m that kind of person… I like to be strong and I’ve also took care of my self. I’ve never asked naebody [nobody] for help. Ever. I help everybody else. But I never ask for help… Now I just don’t want to ask for help. I just go without. I would just stay in” (P2 A). This participant felt that lack of material resources (e.g., access to a car) compounded her struggles undertaking everyday life work (such as carrying heavy shopping).

However, greater support for the work of everyday life that was conducted in private (i.e., within the home), appeared to present less risk of challenge to personal and moral identities. One participant explained that although the initial introduction of mobility aids around the home had been difficult to accept (because of what it meant to her as a relatively young woman), she had been able to adapt well to their use in private. She came to embrace the greater support she had been given as everyday life work had become so much easier to perform: “I’ll take it [bath chair], I’ll try it, but I’m not wanting it. She [physiotherapist] said ‘Well try it’. Now, I wouldnae [would not] part with it” (P10 B).

Participants’ accounts of their responses to offers of support that could potentially lessen their struggles with everyday life work, were revealing of their willingness or reluctance to accept help with managing some of the consequences of multimorbidity.

## Discussion

This study was an in-depth qualitative study examining the management of multimorbidity. In this paper, we have focused on one key area: performance and management of everyday life work. Analysis of participants’ accounts of their everyday life work highlight the contexts in which they were struggling, and those they felt they could manage ‘well’. Four key themes characterized the analysis of everyday life work: (i) the symbolic significance of everyday life work to evidence the work of being ‘normal’; (ii) using everyday life work to help manage emotional distress; (iii) how mental health problems impaired everyday life work; and (iv) accepting help for everyday life tasks. Participants’ accounts implicitly reveal the effects of deprivation (which included descriptions of having to manage many problems and having access to few social and material resources), and how these were perceived to exacerbate their struggles to manage, especially when combined with a mental health problem.

The links between multimorbidity, deprivation, and mental illness have been well established quantitatively in epidemiological [2], cohort [27, 28], and general practice-based studies [5, 29]. Multimorbidity in the context of deprivation is associated with lower quality of life, especially in patients below 65 years [8], and the triad of multimorbidity, deprivation, and mental illness massively increases the risk of unplanned hospital admissions, even for admissions deemed potentially avoidable [27]. The findings presented here offer some explanations as to why some patients with multimorbidity, who shared particular characteristics and experiences of depression, struggled to manage ‘well’ in this social context. Our findings also support the views of GPs and practice nurses reported previously [9], which suggested that the problems patients face in managing multimorbidity are exacerbated by deprivation and lack of access to material, social, and personal resources (see also [22]).

Research has previously demonstrated how important it is for patients to feel their health professionals understand the ‘bigger picture’ of their lives, which includes social problems relating to poverty [29]. This, as Barry et al. [30] suggest, would involve paying attention to “the voice of the lifeworld” and not just to the “voice of medicine”. Certainly, understanding the experience of multimorbidity in relation to getting on with everyday life work is likely be helpful for GPs in guiding and supporting self-management but difficult to explore in the context of usual clinic visits lasting up to 10 min. More time to support people with complex problems in difficult circumstances is likely to be necessary [31].

The analysis has also brought to the fore the importance of emotions and their management in relation to everyday life work with chronic illness in the context of deprivation. It is a concern that some participants raised issues about their vulnerability to abuse, arising from visible disabilities, and the consequences this had for them emotionally and on their willingness to engage in everyday life activities that may enhance wellbeing. Recent research has begun to explore emotions in relation to multimorbidity (a much neglected area), documenting, for example, the frustrations many with chronic pain experience in not being able to perform the everyday tasks of life [32], and how patients may oscillate between feeling anxious and feeling strong and able to deal with their illnesses [33]. McCoy (2009), in a study of the everyday work of taking antiretroviral treatment for HIV, found that managing various illnesses relating to HIV required people to perform work to suppress or adjust emotions as well as to manage their medications [34]. The emotional wellbeing of patients with multimorbidity currently seems to be poorly explored and supported by healthcare systems, especially in deprived areas [35–37]. Further research to understand, and better support to manage, difficult emotions in the context of multimorbidity and deprivation is likely to be helpful.

Our study had several limitations. Common to other research in very deprived areas, we had some difficulty in achieving interviews with participants, with 14 participants in the study interviewed, despite us spending a considerable amount of time in cultivating relationships, by telephone and by making visits. People who said they were willing to be interviewed found they could not fit us in because of a range of problems they were facing. Although we were able to include a range of participants in terms of problems they faced, age range, and sex, we are not sure if we have captured the full range of experiences of those with multimorbidity in very deprived areas. We acknowledge that selection of participants by GPs (who identified patients they perceived to need the greatest support) may mean that some of the sample includes participants who struggled the most, although we took steps to ensure there would be wider variation in the sample. We also acknowledge that the experiences of our participants are shaped by their environment and by what is likely to be seen as acceptable in their own particular culture. Respondents in other areas, in other countries, operating in different health systems, may well have different experiences. Nevertheless, the overriding importance of being able to ‘get on with life’ and maintain a sense of self is common to most studies of the experience of chronic illness, and therefore very likely to be generalizable.

One difficulty we have found, in presenting accounts of managing multimorbidity, is that participants do not necessarily present their experiences of managing in relation to ‘multimorbidity’. The difficulties of having to manage many problems were certainly conveyed; indeed, they were a constant reference point within interviews. However, when participants described challenges they faced in everyday life it was common for them to focus on particular problems (e.g., describing the significance of depression or a particular physical disability for performances of everyday life work). It should be noted, therefore, that describing the experience of managing multimorbidity does appear to involve considering the personal impact of individual diseases, and the meaning these have for individuals’ everyday lives, as well as thinking about the impact of the combination of problems as a whole.

This study is one of the first to look in-depth at the complex relationship between self-management of multimorbidity and explore the everyday life work patients performed in the context of areas of extreme socioeconomic deprivation. Most patients who were interviewed struggled with the amount of work required to establish a sense of normalcy in their everyday lives, especially when their multimorbidity included a mental health problem. Future interventions aiming to improve quality of life and wellbeing in patients with multimorbidity living in deprived areas may benefit from an appreciation of the importance of everyday life work in this population.

## Figures and Tables

**Table 1:** Characteristics of participating patients

Patient/practice^*^	Identification^*^	Sex	Age (years)	Problems they had to manage
P1 B	PR	M	53	*Depression*; diabetes; he discussed tackling his obesity; long-term incapacity (14 years)
P2 A	PR	F	62	Osteoarthritis; chronic pain; diabetes; asthma; liver disease; *depression*; housing problems; mother with dementia; family problems (current and past); socially isolated
P3 A	PR	M	60	Chronic obstructive airways disease; diabetes; alcoholism; *depression*
P4 B	PR	F	50	Neuropathy; liver damage; colostomy; urostomy; diabetes; chronic bowel disease; underactive thyroid; leaking heart valve; lump in breast being explored (family history of cancer) (48 medications); caring for husband (also with multimorbidity); *anxiety and depression*
P5 A	PR	F	59	Bronchitis; asthma; arthritis; diabetes; *depression* (she discussed obesity and smoking also); recent loss of spouse
P6 A	PR	F	53	Kidney stones; arthritis; diabetes; hypertension; underactive thyroid; sleep apnoea; *depression*; liver problems. Most of the interview focused on agoraphobia, sexual abuse and difficulties she had with intimate relationships
P7 A	PR	M	54	Diabetes; hypertension; *stress at work*
P8 B	CR	F	53	Cerebral vascular accident and subsequent disability
P9 A	CR	M	50	Diabetes; hypertension; problems with leg following an accident – multiple surgeries**.** *Stress at work* (being treated for *depression*)
P10 B	CR	F	64	Hypertension; chronic pain; osteoarthritis; obesity; blood pressure; stomach ulcer; *depression*. Interview also explored difficult family relationships (current and past), bereavement (loss of her daughter), her experience of living with a violent partner
P11 B	CR	M	52	*Depression*; erectile dysfunction; epilepsy; psoriasis; bereavement (stated refused medication for depression will self-medicate with St John’s Wort). Interview focused on loss of 14 year old daughter, care of his wife who suffered a cerebral haemorrhage, housing problems, and his suicidal feelings
P12 B	CR	M	52	Chronic pain; ischaemic heart disease; stomach problems; *depression*; erectile dysfunction; mobility (spine problems); financial problems with Department of Social Security
P13 A	CR	M	44	Asthma; stomach problems
P14 B	CR	F	52	Crohn’s disease; joint pain

*CR, researcher identified; PR, practice identified.

**Table 2:** Extracts from participants’ accounts that illustrate the symbolic significance of everyday work.

Description	Verbatim extract
The symbolic significance of everyday work to emphasize attempts at ‘normal life’ in the context of limitations	“I’ve got to be doing something. I’ve got to be tidying up. I’ve got to make sure my house is the way I want. No I wouldn’t sit in my own filth, no. I’ve got to do my housework whether it kills me or not.” (P4 B: female, 50 years, MM + depression) “Doing the garden nearly killed me [laughing], but I had to get it done.” (P10 B: female, 64 years, MM + depression)
The importance of everyday tasks to manage symptoms of depression	Staying occupied and active helped to prevent a “mental death”: “When you’re sitting here all day long, day in, day out, and there’s nothing different… that’s why you get depressed, you know… I walk about here thinking ‘what can I do?’ And you can’t do anything to get your mind off it, you know. But if you’ve got something to do then you don’t think of that and the time goes quicker… You get to a point where your mind goes dead… because you’ve got nothing to think about.” (P12 B: male, 52 years, MM + depression)
	Doing household tasks (often assigned to him by his wife) were a helpful distraction from anxieties about his health: “Sitting here you think about everything and you just… Everything… a wee [small] twinge and you’re ‘och [oh] no, what’s happened now’, you know what I mean? Whereas when you’re working you don’t think of these things. You’re working, you’re busy, you’re doing stuff.” (P9 A: male, 50 years, MM + depression)
Difficulties keeping going with everyday life work due to depression, especially in a poor area	“I’ve stopped going out… I’ve stopped doing this and I’ve stopped doing that. But… it is really silly, but that’s in there [indicates head]… I need to erase the last 3 years and just think positive and think forward… That’s a hurdle I just need to get over [it].” (P9 A: male, 50 years, MM + depression)
	“[My area] is a place where they send you to die really. They should put a fence round it. They send you there to forget about you. They had these houses, they built them, they’re cheap and nasty. Everything about them’s cheap. Everything… We’re shoved up here with nae [no] facilities. The nearest facilities to here are the shopping centre basically that you need a car to get to. There’s nae facilities. When I came here there was bowling greens, the football pitches were actually used. There was bowling greens, tennis courts, the shops. There’s nothing now. The nursery school, primary school, church, everything’s been pulled down. It’s stripped bare. There’s nothing left at all eh very poor quality houses.” (P11 B: male, 52 years, MM + depression)
Difficulties accepting help to support everyday life work	“I feel as if people are thinking, ‘poor soul. Look she’s got a stick’… And that kind of stops me [using it].” (P2 A: female, 62 years, MM + depression)
	“I don’t like being in public using a stick… I was using it in here [in the house]. Half of [the area] has got sticks. Right, I know that’s bad me saying that. But when you see them up there and they’re swopping crutches, you know: ‘I’m going to doctor’s, lend us yer [your] crutches’, know what I mean? And it was like, you know, well, I’m not going out there [with crutches].”(P10 B: female, 64 years, MM + depression)

**Box A:**
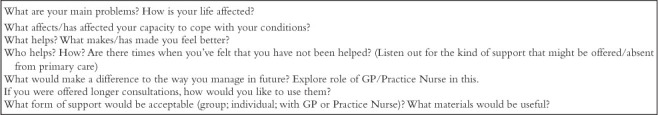
Interview topic guide (designed to open discussion on key areas, responses explored in detail).
